# The Role of Meditation in College Students' Neuroticism and Mental Health

**DOI:** 10.1515/tnsci-2019-0019

**Published:** 2019-04-23

**Authors:** Wenqing Liu, Jiayan Lin

**Affiliations:** 1 Student Affair Office, Wuxi Institute of Technology, Wuxi, Jiangsu 214121, China; 2 School of Art&Design, Wuxi Institute of Technology, Wuxi, Jiangsu 214121, China

**Keywords:** Neuroticism, Contemplation, Health, Meta-analysis, Questionnaire Preparation, Structural equation model

## Abstract

A large number of empirical studies have found evidence that neuroticism is related to personality traits, but no one has integrated the relationship between neuroticism and mental health through meta-analysis. As a personality trait, neuroticism reflects the stable tendency of how individuals experience, feel, evaluate negative emotions and make corresponding behavioural responses. By means of meta-analysis, a preliminary dimension of neuroticism is constructed through an open questionnaire and literature review. On this basis, a preliminary neuroticism questionnaire for college students is compiled. The structural model of College Students' neuroticism questionnaire fits well, and has a high correlation with the neuroticism subscale of the simple version of Big Five Personality Questionnaire, which shows that it has a good structural validity. The positive orientation indicators of subjective well-being, life satisfaction and other mental health indicators were also selected. In addition, self-assessment indicators of physical health were selected. It was found that neuroticism was highly correlated with appeal indicators, indicating that the questionnaire of College Students' neuroticism had good validity.

## 1 Introduction

Contemporary college students shoulder the mission of building socialism with Chinese characteristics and realizing the great rejuvenation of the Chinese nation. Their mental health level is not only related to the stability and development of the future society, but also to the healthy growth of the next generation. Therefore, the mental health of college students has become an important topic in the fields of psychology, education, management, and economics. Research in these fields shows that the mental health of college students is affected by many factors, among which neuroticism is one of the important factors. The core feature of neurotic personality is the tendency to experience negative emotions. This tendency causes individuals to experience more negative emotions and less positive emotions, thus making neuroticism have a negative impact on mental health, manifested as neurotic personality is depression. Susceptibility factors for common mental disorders such as symptoms and anxiety disorders.

As researchers pay more and more attention to the mechanism of sound mental health, the relationship between contemplation and mental health has become a hot research topic. Personality and health are closely related. Although in the final analysis, whether health changes personality or personality affects health is a “chicken or egg first” problem that puzzles psychologists, a large number of studies also reflect that personality and health are highly related. There have been four models about the relationship between personality and mental health: a certain personality may lead to a certain disease, and a certain personality may be caused by a certain disease. Personality may be a perceptual filter personality and illness feedback each other. Neuroticism is one of the universal personality dimensions. This dimension refers to a continuum from extreme emotional instability to emotional stability. People with high neuroticism are highly anxious, moody and easily agitated. At the same time, people with low neuroticism have mild and slow emotional response, and are easy to recover calm, not easy to anxiety, stable and mild, and easy to self-restraint ^[1]^.

Meditation is now regarded as important cognitive factors lead to depression, and is closely related to the individual’s emotional state. When individuals in the social information processing, the meditation of cognitive style tends to lead to repeated thinking of subjective negative feelings, that more durable for depression, poor circulation, meditation and depression will ultimately affect the mental health of the individual.

## 2 Neuroticism and intimacy

Neuroticism is highly correlated with anxiety and depression symptoms, which has been confirmed in the study of normal and clinical samples. Many theories have proposed some models to explain this phenomenon. Some people think that meditation is related to neuroticism and others point out that the mode of meditation response is probably a neurotic cognitive expression. The relationship between neuroticism and mental health of domestic college students was calculated. The homogeneity test was carried out based on the results of meta-analysis. Q=372.4971, df=18, p<0.001. The difference was significant. Random effect model should be used to calculate the average effect value ^[2]^. The meta-analysis of the correlation coefficient between neuroticism and mental health of college students in China is shown in Table 1 below.

**Table 1:** Meta-analysis of the relationship between neuroticism and mental health of domestic college students

Sample number	Research object	Sample size	Correlation coefficient	Lower limit	Upper limit	Z value	P value
1	Freshman	7961	0.680	0.698	0.672	73.953	0.000
2	College Students	1655	0.663	0.630	0.689	32.441	0.000
3	Art undergraduate	512	0.460	0.324	0.526	11.220	0.000
4	Hunan University	1800	0.630	0.624	0.657	31.429	0.000
5	Military school	469	0.655	0.623	0.704	16.924	0.000
6	Military Academy	429	0.474	0.310	0.544	10.634	0.000
7	College Students	624	0.567	0.519	0.636	16.773	0.000
8	Old college student	322	0.540	0.415	0.631	11.303	0.000

Neuroticism is not only closely related to depression and anxiety, but also to physical fitness. It is also closely related to life satisfaction. Some studies have found that there is a significant negative correlation between neuroticism and life satisfaction ^[3]^. Further studies have found that neuroticism is a negative predictor of life satisfaction. In short, the higher the individual’s neuroticism, the lower the life satisfaction. Calculate the relationship between introversion and extroversion and mental health of college students at home and abroad, and test the homogeneity of the results, Q=170.9358, df=17, p<0.001. The difference is significant. It shows that the data results come from different populations, and the random effect model should be used to calculate the average effect value ^[4]^. The meta-analysis of the correlation coefficient between introversion and extroversion and mental health of college students at home and abroad is as follows:

As an important risk factor in psychopathology, neuroticism is closely related to depression. It was found that all the diagnostic groups scored better in neuroticism. Anxiety disorder had the closest relationship with neuroticism, followed by depression disorder, and substance abuse disorder had the weakest relationship. However, depression increased faster in high neuroticism individuals than in low neuroticism individuals. In short, the higher the neuroticism level of individuals, the more depressive emotions they experience, the higher the risk of depression, and the higher the risk of recurrence of depression after cure ^[5]^.

## 3 The relationship between contemplation and mental health

Many individuals tend to adopt a negative coping style when they realize that the target state of the self is different from the current state, which is, seeking the result and value meaning by repeatedly thinking about the same subject content. The response method often does not help the individual to solve the problem, but will deepen the individual’s level of depression. This coping style is contemplation.

Mediation effect test must satisfy the following conditions: independent variables can significantly predict intermediary variables and dependent variables; after controlling independent variables, intermediary variables can significantly predict dependent variables; after controlling intermediary variables, the predictive effect of independent variables on dependent variables is significantly reduced ^[6]^. If the independent variable predicts the dependent variable significantly after controlling the intermediary variable, there will be some intermediary effect. If the independent variable does not predict the dependent variable significantly after controlling the intermediary variable, there will be a complete intermediary effect.

**Table 2:** Meta-analysis of neuroticism and mental health in foreign countries

Sample name	Sample size	Correlation coefficient	Lower limit	Upper limit	Z value	P value
John Maltby	320	0.137	0.028	0.243	2.455	0.014
Paula Williams	135	-0.260	-0.411	-0.095	-3.057	0.002
Corina Greven	1038	-0.290	-0.345	-0.233	-9.605	0.000
R.B.Willkinson	404	-0.350	-0.433	-0.261	-7.318	0.000
Adrian Furnham	120	-0.150	-0.321	0.030	-1.635	0.102
Mary E. Stewart	338	-0.250	-0.347	-0.147	-4.675	0.000
Liang-Lin Chien	1898	-0.190	-0.233	-0.146	-8.373	0.000
James P. David	96	-0.060	-0.257	0.142	-0.579	0.562

**Table 3:** Fitting index of the structure of college students’ neurotic questionnaire

χ2/df	GFI	AGFI	CFI	PNFI	PCFI	RMSEA
3.965	0.873	0.854	0.833	0.685	0.731	0.057

### 3.1 Correlation analysis of depressive symptoms, neurotic personality and contemplation

**Figure 1:**
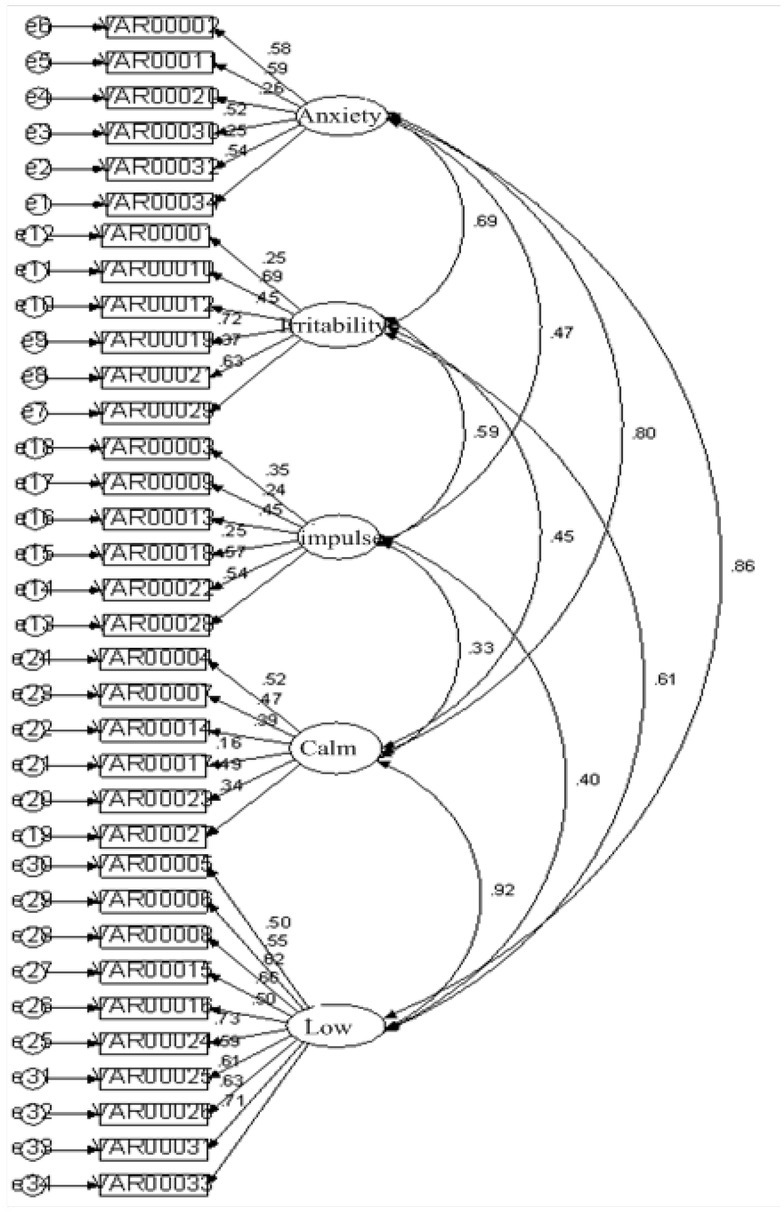
Confirmatory factor analysis path coefficient graph of neurotic questionnaire

The predictive effect of neurotic personality on depressive symptoms was significantly reduced. The standardized regression coefficient of neurotic personality on depressive symptoms decreased from 0.601 before the introduction of mediating variables to 0.414 after the introduction of mediating variables, indicating that there was mediating effect ^[7]^. Fitting degree between the constructed five-factor neuroticism structure model and the measured data. There are many fitting indexes for confirmatory factor analysis, such as chi-square freedom ratio_2/df, RMSEA of approximate error, GFI of goodness of fit, AGFI of absolute fitting index, CFI of comparative fitting index, PNFI and PCFI of simple fitting index. If 2/df is less than 5, the model fits well. The closer RMSEA is to 0, the better RMSEA is to 0. 1, which means that the model fits well. The values of GFI, AGFI and CFI are between 0 and 1. Generally speaking, the model fits well when they are larger than 0.85. Simple fitting index PNFI and PCFI if greater than 0.5 also shows that the model fits well. The fitting index of the model is shown in the following table:

As can be seen from the table, the model 2/ df is less than 5, RMSEA is far less than 0.1, GFI, AGFI and CFI are all greater than 0.85, PNFI and PCFI are all greater than 0.5, except CFI is close to 0.85. It shows that the model fits well and the five-factor neuroticism model has good structural validity, as shown in Figure 1.

### 3.2 The relationship between contemplation and anxiety

As a stable coping style, meditation makes individuals tend to think continuously without solving problems in the face of sad emotions. The reaction style theory that leads to depression is pointed out that the meditation of negative events will increase the depression of individuals and be negative. Cognitive influences amplify negative emotions. To more fully validate this theory, Nolen-Hoeksema’s longitudinal study of adolescents found that meditation can predict depressive symptoms and drug abuse behaviour findings that anger meditation makes individuals More attention to the possible outcomes after anger, and thus unable to extricate themselves, is easy to provoke the individual’s bad mood. The results show that meditation is not only a symptom of depression, but also an important factor in predicting the persistence of an individual’s negative emotions.

**Figure 2:**
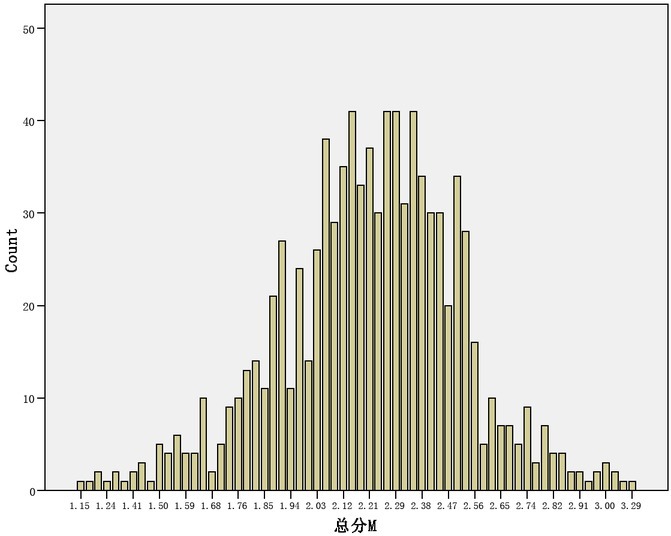
Relationship between score distribution of neurotic score and anxiety

There is also a close relationship between meditation and anxiety. The results show that meditation is significantly correlated with anxiety. A sample of college students has been studied. The results show that meditation, compulsive meditation and reflection are strongly correlated with anxiety. Meditation is not only related to anxiety, but also to social anxiety. Individuals with high meditation level will have more inadaptable negative thoughts, fewer problem solving behaviours, fewer Du will support and more social friction, resulting in a higher level of anxiety experienced by individuals. In order to investigate whether the scores of contemplation and anxiety of college students conform to the normal distribution, the distribution of the scores of contemplation and anxiety of college students is shown in Figure 2.

**Figure 3:**
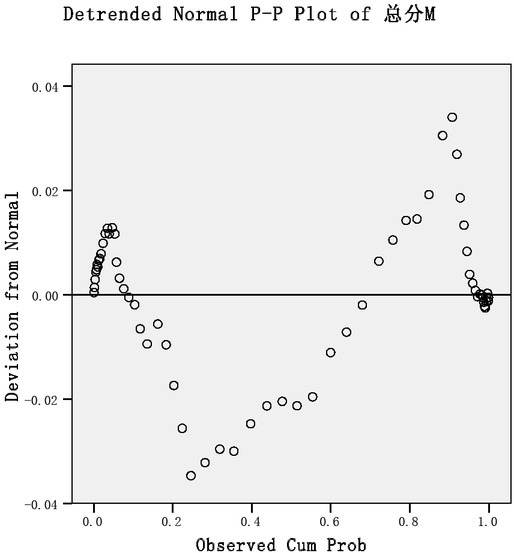
Neurogenic total score distribution normal distribution test castration PP diagram

From the above figure, we can see that the total score of neuroticism and various dimensions of college students are basically low, which shows that the emotional stability of college students is better basically. From various dimensions, college students are more likely to experience anxiety ^[9.^ It can be seen that the emotional stability of college students is basically good, and in terms of the tendency to experience negative emotions, anxiety is more than depression. To test whether the sample is normal distribution, the sample is generally tested by castrated P-P chart. As shown in Figure 3, the castrated P-P chart of the total neuroticism score of college students is as follows:

From the graph, we can see that the normal distribution test of each dimension shows that the scores of each dimension also obey the normal distribution.

### 3.3 Relationship between meditation and life satisfaction

Meditation training can improve positive emotions and life satisfaction, reduce negative emotions, and effectively improve the subjective well-being and psychological well-being of individuals. It can relieve stress, improve the ability of emotion regulation, effectively improve the level of anxiety and depression of individuals, improve the level of alpha wave of EEG, reduce the level of EMG and heart rate, and significantly improve the increase of my electricity and heart rate caused by the activation of the sympathetic nervous system fast. Mindfulness meditation training reduces stress, regulates emotions, enhances self-acceptance and pursuit of life goals, promotes inner growth, and establishes good relationships with others is one of the potential mechanisms for enhancing individual subjective well-being and psychological wellbeing.

Domestic and foreign studies have not explored the role of meditation in life satisfaction. Because anxiety and depression are both negative emotions, the relationship between meditation and anxiety has also attracted the attention of researchers. Life satisfaction is the cognitive component of subjective well-being, because meditation can aggravate negative emotions, send some negative emotions, negative memories and negative thoughts, which may have a negative impact on individual life satisfaction ^[10]^. Therefore, reveal the relationship between meditation and life satisfaction, and put forward reference suggestions to improve individual life satisfaction. Stepwise regression analysis was carried out with the scores of neuroticism and subjective well-being as independent variables. The variance of 24.4% was explained.

**Table 4:** Stepwise Regression Analysis of Contemplation on Subjective Well-being

Mentality	R2	after adjustment of R2	R2 change amount	F value	βvalue	t value
Low sensitivity	.218	.217	.218	125.637***	-.347	-6.480***
Low sensitivity	.239	.236	.021	70.557***	-.188	-3.510***
Calm and open-minded	.249	.244	.010	49.643***	-.287	-4.903***
Low sensitivity	.201	.239	0.12	390.123***	-.187	-3.516***
Calm and open-minded	.203	.210	0.45	70.762***	-.119	-2.487***

Among them, low-key sensitivity was the most predictive, and its individual explanation was 21.7%. The results of regression analysis of neuroticism on subjective well-being are as follows:

With the scores of neuroticism and overall satisfaction as independent variables, stepwise regression analysis was carried out. The three dimensions included in the equation were low sensitivity, calm and open-minded, and impulsive excitement.

## 4 The intermediary mechanism of neuroticism affecting mental health

Neuroticism has indirect effects on depression through meditation mediation W and chain mediation of self-esteem and optimism, and the chain mediation effect of self-esteem and optimism is greater than that of meditation; meditation plays a complete mediation role between neuroticism and anxiety. Self-esteem plays a complete mediating role between neuroticism and life satisfaction. The influence of neuroticism on life satisfaction is mediated by self-esteem.

### 4.1 Neurotic intervention in meditation

Meditation is an individual’s cognitive style, which plays an important role in depression, anxiety disorders and other mental disorders, as well as vascular diseases, other chronic diseases and other physiological diseases. Therefore, reducing the level of individual meditation can improve the level of individual physiology and physical health. Effective therapies for reducing meditation include the following: attention diversion, mindfulness training, including mindfulness decompression therapy and mindfulness cognitive behavioural therapy. This non-critical acceptance may be one of the internal mechanisms for reducing meditation.

### 4.2 The mechanism of neuroticism on positive mental health

**Figure 4:**
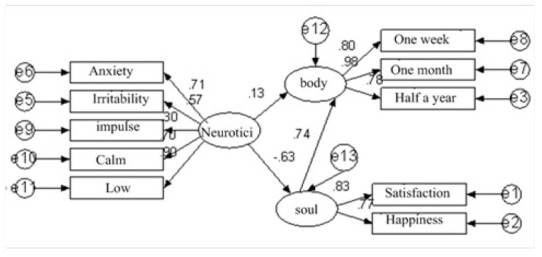
Relationship between neuroticism and physical and mental health

With meditation as the mediating variable, we found that neuroticism mediates between neuroticism and life satisfaction through meditation. Compared with the complex mediating mechanism between neuroticism, depression and anxiety, the mediating mechanism between neuroticism and life satisfaction is relatively simple. This shows that neuroticism is a negative and rational trait, and the mechanism of its influence on negative health indicators such as depression and anxiety is complex, as shown in Figure 4. The impact on health indicators such as life satisfaction is relatively simple. The complex mechanism of neuroticism affecting health was discussed. It was found that the mechanism of neuroticism affecting negative health indicators such as depression and anxiety was complex, and the mechanism of neuroticism affecting health indicators such as life satisfaction was relatively simple ^[11]^. This shows that in the process of psychological intervention, we should improve life satisfaction, at the same time, focus on eliminating negative health problems such as depression and anxiety.

**Table 5:** Stepwise regression analysis of mental health on physical health

	Non-standardized coefficient		Standardization coefficient	t value	p value
(Constant)	9.846	.947	.156	10.393	.000
Subjective well-being	.585	.132	.218	4.441	.000
Satisfaction	.343	.039	.436	8.874	.000

Using AMOS 7.0 constructs the structural equation model of neuroticism, mental health and physical health. As shown in the figure above, the fitting index of the model is shown in Table 6.

**Table 6:** Fitting index of neuroticism and physical and mental health relationship model

χ2/df	GFI	AGFI	CFI	NFI	TLI	RMSEA
4.138	0.947	0.909	0.953	0.939	0.934	0.083

From the above table, we can see that the fitted indexes of neuroticism and physical and mental health model are good, which shows that the model is reasonably constructed. Meditation, that is, compulsive meditation and reflection, partially mediates the relationship between neurotic personality and depressive symptoms.

## 5 Conclusion

Contemplation is a complex mode of thinking. With the deepening of deep thinking research, more and more researchers have carried out multi-level analysis on it, but our research in the field of contemplation is still in the preliminary stage. Existing contemplative research Focusing on the related theories, meditation and the relationship between gender, age, negative emotions and mental health, but there are few studies on the influence mechanism and neurobiological basis of meditation, the measurement tools are relatively simple, and have not yet formed unified meditation definition.

Neuroticism not only has a direct impact on anxiety, but also has an indirect impact on anxiety through the chain mediation between meditation and optimism. Meditation plays a mediating role between neuroticism and depression, and meditation plays a chain mediation role between neuroticism and depression. Meta-analysis showed that neuroticism was highly correlated with the indicators of mental health symptoms, and the results were relatively consistent. As a multi-level and multi-dimensional personality trait, neuroticism includes such dimensions as depression sensitivity, calm and open-minded, anxiety and anxiety, irritability and hostility, impulse and excitement. After introducing meditation as a new mediating variable, neuroticism has a direct impact on depression, anxiety and life satisfaction. Neuroticism has indirect effects on anxiety through mediation of meditation and chain mediation of meditation and optimism, that is, meditation plays a complete mediation role between neuroticism and anxiety, and meditation plays a complete mediation role between neuroticism and life satisfaction.
